# Plant Cell Wall as a Key Player During Resistant and Susceptible Plant-Virus Interactions

**DOI:** 10.3389/fmicb.2021.656809

**Published:** 2021-03-12

**Authors:** Edmund Kozieł, Katarzyna Otulak-Kozieł, Józef Julian Bujarski

**Affiliations:** ^1^Institute of Biology, Department of Botany, Warsaw University of Life Sciences – SGGW, Warsaw, Poland; ^2^Department of Biological Sciences, Northern Illinois University, DeKalb, IL, United States

**Keywords:** plant viruses, cell wall remodeling, defense response, hypersensitive reaction, ultrastructure

## Abstract

The cell wall is a complex and integral part of the plant cell. As a structural element it sustains the shape of the cell and mediates contact among internal and external factors. We have been aware of its involvement in both abiotic (like drought or frost) and biotic stresses (like bacteria or fungi) for some time. In contrast to bacterial and fungal pathogens, viruses are not mechanical destructors of host cell walls, but relatively little is known about remodeling of the plant cell wall in response to viral biotic stress. New research results indicate that the cell wall represents a crucial active component during the plant’s response to different viral infections. Apparently, cell wall genes and proteins play key roles during interaction, having a direct influence on the rebuilding of the cell wall architecture. The plant cell wall is involved in both susceptibility as well as resistance reactions. In this review we summarize important progress made in research on plant virus impact on cell wall remodeling. Analyses of essential defensive wall associated proteins in susceptible and resistant responses demonstrate that the components of cell wall metabolism can affect the spread of the virus as well as activate the apoplast- and symplast-based defense mechanisms, thus contributing to the complex network of the plant immune system. Although the cell wall reorganization during the plant-virus interaction remains a challenging task, the use of novel tools and methods to investigate its composition and structure will greatly contribute to our knowledge in the field.

## Introduction

Plant viruses are highly diversified and cause enormous alterations and deformations inside a plant host cell (Gergerich and Dolja, 2006). Their genome, which has the form of dsDNA, ssDNA, dsRNA, or ssRNA, is surrounded by a capsid consisting of coat protein molecules (Gergerich and Dolja, 2006; Hull, 2014). Generally, the viral genome encodes genetic information about proteins that play a crucial role in the induction and nondisturbed maintenance of viral infection *via* local/systemic transport (Hull, 2014). Thus, plant viruses are active only inside the host cell and induce multilevel changes in its internal system during infection.

Due to their constant exposure to a wide range of pathogenic microorganisms, plants have evolved both constitutive and inducible defense mechanisms (Underwood, 2012). Constitutive defenses of plants encompass physical barriers, such as waxy epidermal cuticles or cell walls on the surface, which prevent the penetration of pathogens. On the other hand, inducible defense responses are activated in plants upon the recognition of potential pathogens by surveillance mechanisms (Jones and Dangl, 2006). Therefore, it can be considered that plants do not remain as “static components” during their interaction/contact with a viral pathogen. Rather, they induce complex systems of response to the invading virus (Mandadi and Scholthof, 2013). The speed, strength, and level of effectiveness of this response determines the susceptibility or resistance of a plant host. The cell wall is not only an inherent structural component in plant cells but also acts as an important “contact platform” during plant-pathogen interactions.

The structural polysaccharides present in the cell wall maintain the shape and size of the cell, while also providing mechanical strength required to endure extrinsic stresses and to preserve the inner turgor (Bacete et al., 2018; Voiniciuc et al., 2018). Numerous reviews have described that the polysaccharide (cellulose) and the non-saccharide fraction (lignin) of the plant cell wall play the main role in developmental, defense, and bioconversion processes (Chen et al., 2018a; Liu et al., 2018; Meents et al., 2018). According to the commonly known, classic model of cell wall structural networks, the surface of cellulose microfibrils is interconnected with hemicellulose fibers enclosed by a pectin matrix, which determines the flexibility or stiffness of the cell wall (Cosgrove, 2018). Broxterman and Schols (2018) postulated that the two components are linked by distinctive and strong covalent interactions in the primary cell wall architecture. Moreover, cell wall polysaccharides serve as a repository of molecules that are involved in intercellular signaling and that elicit the immune response to microbial invasion (Cosgrove, 2018).

A majority of previous studies have focused on the changes occurring in the plant cell wall and put forth different models of plant-pathogen interactions for organisms such as bacteria, nematodes, and fungi, but not viruses. Research on the engagement of the plant cell wall is highly focused on the changes occurring in plasmodesmata during the regulation/blockade of virus cell-to-cell transport. The results revealed the role of β-1,3 glucanase in controlling plant viruses during local and systemic transport (Beffa and Meins, 1996; Iglesias and Meins, 2000). However, recent studies indicate that the frequency of active remodeling and rebuilding of the cell wall, taking place in response to viral infection, is higher than assumed (Raggi, 2000; Ding et al., 2008; Park et al., 2017; Chen et al., 2018b,c; Otulak-Kozieł et al., 2018a). The process of wall rebuilding differs in susceptible and resistant plant hosts (Otulak-Kozieł et al., 2018b, 2020). This review presents the current knowledge about the role of plant cell wall in susceptible and resistance responses of plants to viruses and summarizes the potential fields of future research for a better understanding of this aspect of plant-pathogen interactions.

## Cell Wall in Viral Infection of Susceptible Host

During host-pathogen interactions, plant viruses build a complex system to evade or suppress the defense response of the plant (Garcia-Ruiz, 2018, 2019). If the virus manages to overcome the defense/response system, the plant host becomes susceptible to infection (Jin et al., 2018). The cell wall is a vital element of the host reaction system. Interestingly, novel comparative transcriptome profiling analysis as well as microarray gene expression analysis carried out in susceptible and resistant host plants after the viral infection has shown that the first target in the host cells is the cell wall (Shimizu et al., 2007, 2013; Zheng et al., 2013; Allie et al., 2014). The earlier reports demonstrating the involvement of the cell wall were based on tobacco mosaic virus (TMV) infection in susceptible tobacco plants (Beffa and Meins, 1996; Bucher et al., 2001). The authors of these studies demonstrated that cell wall-associated proteins and enzymes were involved in controlling the virus cell-to-cell transport. One of the main cell wall-associated polysaccharides—callose—was found to be extremely important in the specific control of TMV transport. The presence/distribution of this protein in the apoplast area was directly regulated by the ratio of two enzymes: callose synthase (which catalyzes the callose synthesis) and β-1,3 glucanase (which hydrolyzes callose; Kauss, 1996). In response to TMV (and other plant viruses), plants increase the synthesis and deposition of callose which can be observed near and inside the plasmodesmata. Callose deposited inside the plasmodesmata forms a physical barrier decreasing the size exclusion limit and blocking the cell-cell transport. However, this response could be frequently counteracted by some viruses, including cucumber mosaic virus (CMV), potato virus X (PVX), or TMV, through a mechanism that is rather universal among viruses. The class II β-1,3 glucanase includes the pathogenesis-related protein PR-2, which acts as an important host cellular factor governing the movement of the virus (Beffa and Meins, 1996). Iglesias and Meins (2000) clearly showed that infection by TMV enhances PR-2 activity in tobacco while also increasing the mobility and distribution of plasmodesmata, thus facilitating virus movement. Therefore, degradation of the callose physical barrier (due to increased deposition of β-1,3 glucanase) is essential for the maintenance of viral movement in a susceptible host (Bucher et al., 2001). The increased deposition of β-1,3 glucanase allows TMV and other viruses to overcome the natural blocking mechanism and promotes pathogen transportation. The changes of the cell wall are associated not only with plasmodesmata but also with apoplasts. The South African cassava mosaic virus is the first example reported to have this finding. Allie et al. (2014) postulated that susceptible cassava genotype T200 infected by SCMV induced mRNA transcripts of several components including proteins belonging to pectin lyase superfamily or plant invertase/pectin methylesterase inhibitor superfamily, which are responsible for the degradation of the plant cell wall. Similar transcriptome data were acquired by Zheng et al. (2013). The authors indicated that a susceptible rice cultivar (cv. Fengjin) responded to infection by rice stripe virus (RSV) by downregulating four genes that code for glycine-rich, cell wall structural protein, and a gene coding for cellulose synthase. Their results suggest that RSV and SCMV could structurally modify the cell wall by specific and complex regulation of cell wall structural proteins.

Changes occurring within the ultrastructure in a susceptible plant host were first reported for potato virus Y-NTN (PVY^NTN^) infection by Otulak-Kozieł et al. (2018a). We ascertained that PVY^NTN^ could be folded into a loose structure. Moreover, they were associated with paramural bodies within the plasmodesmata in the affected portions of cell wall and with virus cytoplasmic inclusions. These changes correlated with alterations in the quantity and distribution of PR-2 and with the catalytic subunits of cellulose synthase (CesA4). Accumulation of PR-2 was noticeably elevated in the susceptible potato. Compared to mock-inoculated plants, the level of CesA4 was decreased in hosts that were susceptible to PVY^NTN^. This infection also changed the distribution of both proteins inside the cells of compatible plant hosts. In the infected susceptible potato, PR-2 was more frequently accumulated in the cell wall and vacuoles compared to the healthy plants. Moreover, in susceptible plants, CesA4 was deposited in the cell wall, plasma membrane, and endoplasmic reticulum. This suggests that both the decrease in CesA4 and increase in PR-2 determined the susceptibility of potato (Otulak-Kozieł et al., 2018a), most likely by enabling cell-to-cell movement of the virus.

**Figure 1:**
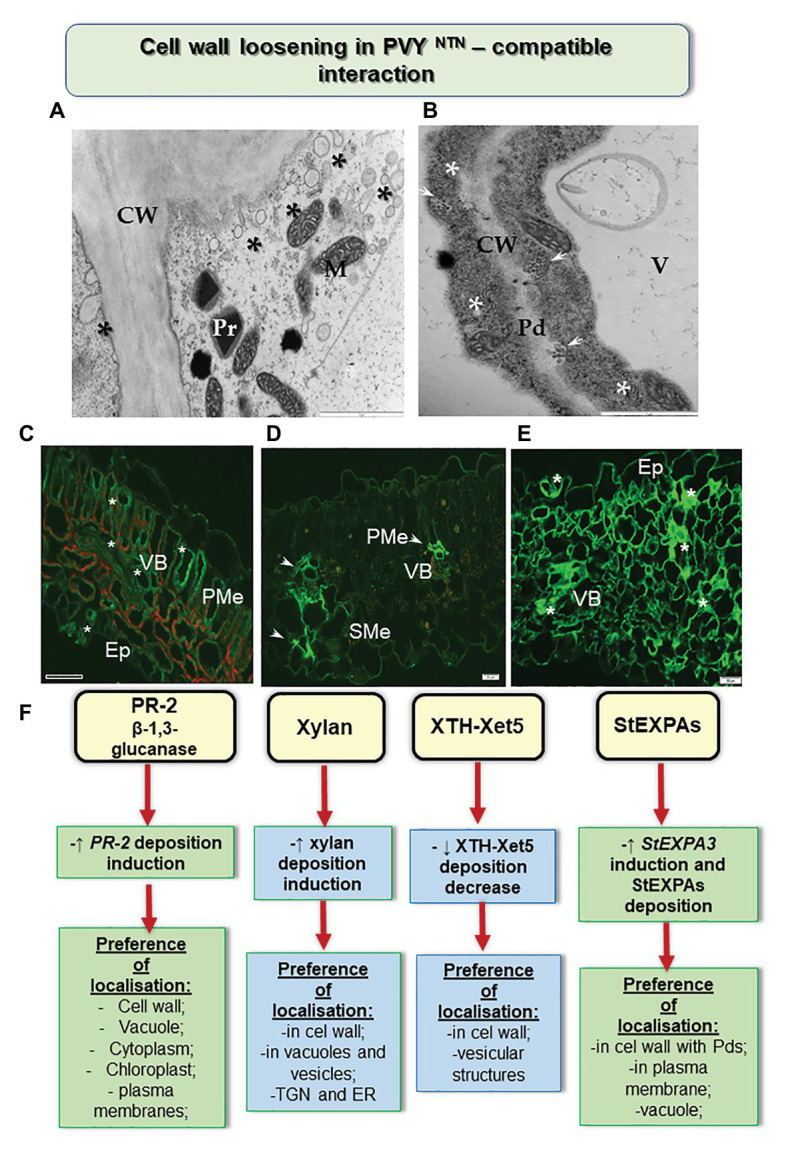
Cell wall loosening in susceptible potato reaction to PVY^NTN^. **(A)** Loosening of the cell wall structure with the distribution of vesicles (*) as shown by transmission electron microscopic analyses. Bar: 2 μm. **(B)** Paramular bodies (arrows) connected with plasmodesmata in association with viral cytoplasmic inclusion (*). Bar: 2 μm. **(C)** Green fluorescence of PR-2 protein (*) observed in the vascular bundle, palisade mesophyll, and epidermis with stomata in susceptible potato leaf. Bar: 200 μm. **(D)** Fluorescence of xyl-1/xyloglucan observed in vascular bundle (arrows; xylem and phloem) and spongy mesophyll cells after 14 days of inoculation with PVY^NTN^. Bar: 20 μm. **(E)** StEXPA signal observed in the leaf tissues at 30 days of post inoculation—signals with the highest intensity (*) were observed in cell wall and symplast of necrotizing mesophyll cells, xylem tracheary elements, and stomata. Bar: 50 μm. **(F)** Schematic representation of the distribution changes of selected cell wall components in susceptible potato response to PVY^NTN^. CW, cell wall; Ep, epidermis; M, mitochondria; Pd, plasmodesmata; PMe, palisade mesophyll; Pr, peroxisome; SMe, spongy mesophyll cells; V, vacuole; VB, vascular bundles. (Based on Otulak-Kozieł et al., 2018a,b, 2020, modified).

The results reported by Otulak-Kozieł et al. (2018b) shed some light on susceptible potato-PVY interactions (Figure 1). The authors studied the *in-situ* dissemination of various hemicellulosic cell wall matrix components during the interactions of susceptible and hypersensitive potato with PVY^NTN^. Xyloglucan was identified as the major hemicellulose of the primary cell wall and was associated with the changes initiated by the pathogen (Pauly et al., 2013). Lotan and Fluhr (1990) and Bacete et al. (2018) postulated that xyloglucan metabolism is linked with cell wall expansion, and thus influences the infection by a pathogen. Organisms such as viruses disrupt the plant cell wall, as well as inducing β-1,4 xylanase, resulting in the production of endoxylanases. We ascertained that the cell wall loosening process occurs along with enhanced deposition of xylan in the event of susceptible interactions after infection of potato with the potyvirus. The cell wall of plants consists of several enzymes capable of modifying polysaccharides, of which xyloglucan endotransglucosylase/hydrolase (Xet, XTH) is of importance, as it is essential for wall architecture and elongation (Fry, 1995). It is commonly known that during the development of a plant, XTH/Xet is involved in cell wall loosening and expansion as well as improving its rigidity during infection by a pathogen (Rose et al., 2002). The PVY inoculation significantly redirected the deposition of xylan-1/xyloglucan and xyloglucan xyloglucosyl transferase (XTH-Xet5), compared to mock-inoculated tissues (Otulak-Kozieł et al., 2018b). Moreover, immunogold localization showed that Xet5 was dominant in cell wall and its nearby vesicles in the host susceptible to infection. The involvement of xyloglucan endotransglucosylases was also identified in the case of interactions of hosts with potato leafroll virus (DeBlasio et al., 2015) and with papaya meleira virus (Rodrigues et al., 2011).

The rebuilding of the cell wall during the growth and development of the plant cell or during the interaction of a plant with nonviral pathogens in the area associated with extensins and expansins, has been postulated by Marowa et al. (2016). However, Yang et al. (2007) stated that in *Arabidopsis* the patterns of expression of cell wall-related genes, including pectin methylesterase 3 (PME3), expansin 10 (EXP10), and xyloglucan transferase 6 (XTH6), varied based on the areas in which the samples were harvested. The authors also noticed a reduction in mRNA transcript accumulation after infection by turnip mosaic virus (TuMV). In addition, they found that the genes PME3, XTH6, and EXP10 were activated against TuMV after 10 days of inoculation. Moreover, Chen et al. (2018c) proposed that EXPA4 overexpression accelerated the replication of TMV and the development of symptoms in tobacco. Similar conclusions could be drawn based on studies analyzing the role played by extensins and expansins during infection of susceptible potato by PVY^NTN^ (Otulak-Kozieł et al., 2020). Otulak-Kozieł et al. (2020) indicated a remarkable induction of *StEXPA3* and slight induction of *StEXT4* during a susceptible response (Figure 1). In addition, the process of cell wall loosening occurred together with an increased deposition of StEXPA glycoproteins and hydroxyproline-rich glycoproteins (HRGPs) in PVY^NTN^-susceptible potato. Interestingly, the StEXPA signal gradually increased in susceptible PVY^NTN^-infected potato, unlike the resistance response which is often found within 1–7 days of inoculation in vascular tissues and 14–30 days of inoculation in most of the leaf tissues. In addition, StEXPAs were detected in the symplast of cells, mainly in the epidermal and stomata regions and in vascular bundles, especially in cell walls (Otulak-Kozieł et al., 2020). Furthermore, we showed the presence of StEXPAs in the plasmodesmata during the susceptible reaction as well as near the cytoplasmic inclusions of PVY—distinctive for the Potyvirus group. However, based on the results obtained from the microarray analysis of the expression of cell wall-related genes following RSV infection, Shimizu et al. (2007) reported marked suppression of other groups of extensins—proline-rich glycoproteins and glycine-rich glycoproteins. They concluded that different types of extensins could be up- or downregulated during interaction with different plant viruses.

## Plant Cell Wall and the Plant-Virus Resistance Response

Defense activation during the identification of exogenous or endogenous signals—known as pathogen-associated (PAMPs) and damage-associated molecular patterns (DAMPs), respectively—is the key function of innate immunity in plants (De Lorenzo et al., 2019). Damage detection is essential for plant cell survival. Mechanical damage and infections disturb the homeostatic cellular processes, which are recognized as a threat by plants (De Lorenzo et al., 2019). Perception of “damaged self” occurs independently of the infecting organism. Hence, the induced response may not be particular to a given pathogen (Heil and Land, 2014; De Lorenzo et al., 2019). DAMPs might result from the damage caused to cell structures by injuries and consequently developmental breaks; therefore, they not only protect against infection but also play a crucial role in processes that are not related to pathogens, including tissue injuries and repair (De Lorenzo et al., 2018). DAMPs can be exemplified by oligosaccharides discharged by the cell wall. The structure of the plant cell wall is closely observed, upon cell wall remodeling. Moreover, it is significantly modified by mechanical injuries or by infection (Ferrari et al., 2013; Bellincampi et al., 2014; Hamann, 2015; De Lorenzo et al., 2019). According to the analyses of Benedetti et al. (2015) and Gramegna et al. (2016), the products resulting from the breakdown of homogalacturonan (HG), such as oligogalacturonides (OGs), are treated as DAMPs produced against microbial attack and as a local signal to repair mechanical damages. Moreover, Savatin et al. (2014) stated that during an infectious event, OGs are produced by the effect of enzymes that degrade microbial pectin. The defense responses are actively induced by OGs consisting of 10–15 residues, whereas shorter oligomers reveal lower activity, according to Davidsson et al. (2017). Cabrera et al. (2008) highlighted that the OGs function maximally when involved in calcium-mediated intermolecular ionic interactions that render these compounds with a conformational state referred to as “egg boxes.” Moreover, the extent of HG methyl-esterification or acetylation, which differs in organs during the development of plants, may decide the characteristics of OGs released and their biological activity. As analyzed by Benedetti et al. (2015), average OG accumulation triggers an appropriate and stable immune response, whereas excess OGs may induce hyper immunity, affecting growth and ultimately leading to cell death.

The PAMP and DAMP response pathways associated with the cell wall were not precisely recognized for plant viral infections. In fact, numerous results indicate that the cell wall is actively modified during a plant resistance response against viral pathogens. Years of plant-virus co-evolution did not only lead to susceptibility but also lead to resistance. Generally, resistance, or high resistance (associated with hypersensitive reaction), results from well-developed and effective defense mechanisms induced to prevent or limit the damage caused by infection by a viral pathogen (Soosaar et al., 2005). Plant resistance genes confer hosts with resistance against various pathogens, including viruses (Soosaar et al., 2005; Hull, 2014). The defense response initiated as a result of the recognition of a specific virus is stereotypical, and the associated cellular and physiological features have been well described by several authors (Soosaar et al., 2005; Hashimoto et al., 2016; Kumar, 2019).

The attribution of the cell wall to the resistance response against plant viruses is a newly emerging concept. Generally, several cell wall mutants or plants treated with cell wall biosynthesis inhibitors have been shown to exhibit a gamut of immune response, including the expression of defense genes and the accumulation of defense compounds, but it is rather limited in the case of plant-virus interactions (Hamann, 2015). The first investigations of plant-virus interactions concerned plasmodesmata. Inhibition of β-1,3 glucanase enzyme led to an elevated deposition of callose in some parts of the cell wall, thereby leading to a reduction in size exclusion limit. As a result, both short- and long-distance transport of CMV, TMV, PVX, and tobacco necrosis virus was reduced (Iglesias and Meins, 2000; Peña and Heinlein, 2012). Plants with suppressed β-1,3 glucanase restricted the access of plant viruses to a limited number of cells, in which it was possible to induce programmed cell death, thus repressing or even stopping virus infection (Iglesias and Meins, 2000). A similar limitation, characteristic of TMV, was observed in potato with a hypersensitive response (HR) against PVY^NTN^ (Otulak-Kozieł et al., 2018a). In this reaction, intense callose deposits were immuno-localized in plasmodesmata, accompanied by lower PR-2 deposition, as compared to the compatible interaction. In this case, PR-2 was mainly transported and deposited in vacuoles rather than in the cell wall. Apparently, potato actively redistributed this protein in the presence of the virus. In addition, resistant tobacco plants (VAM) that were infected with PVY responded with increased deposition of PR protein-related transcripts (Chen et al., 2016). On the other hand, the data presented by Lionetti et al. (2014) indicated the role of pectin methylotransferase inhibition in viral transport and plant resistance in *Arabidopsis thaliana* and *Nicotiana tabacum*. The authors showed that the overexpression of genes encoding pectin methylesterase inhibitors in *Nicotiana tabacum* and *Arabidopsis thaliana* from *Actinidia chinensis* generated some level of resistance and limited the transport of two different plant viruses namely TMV and turnip vein-clearing virus.

**Figure 2:**
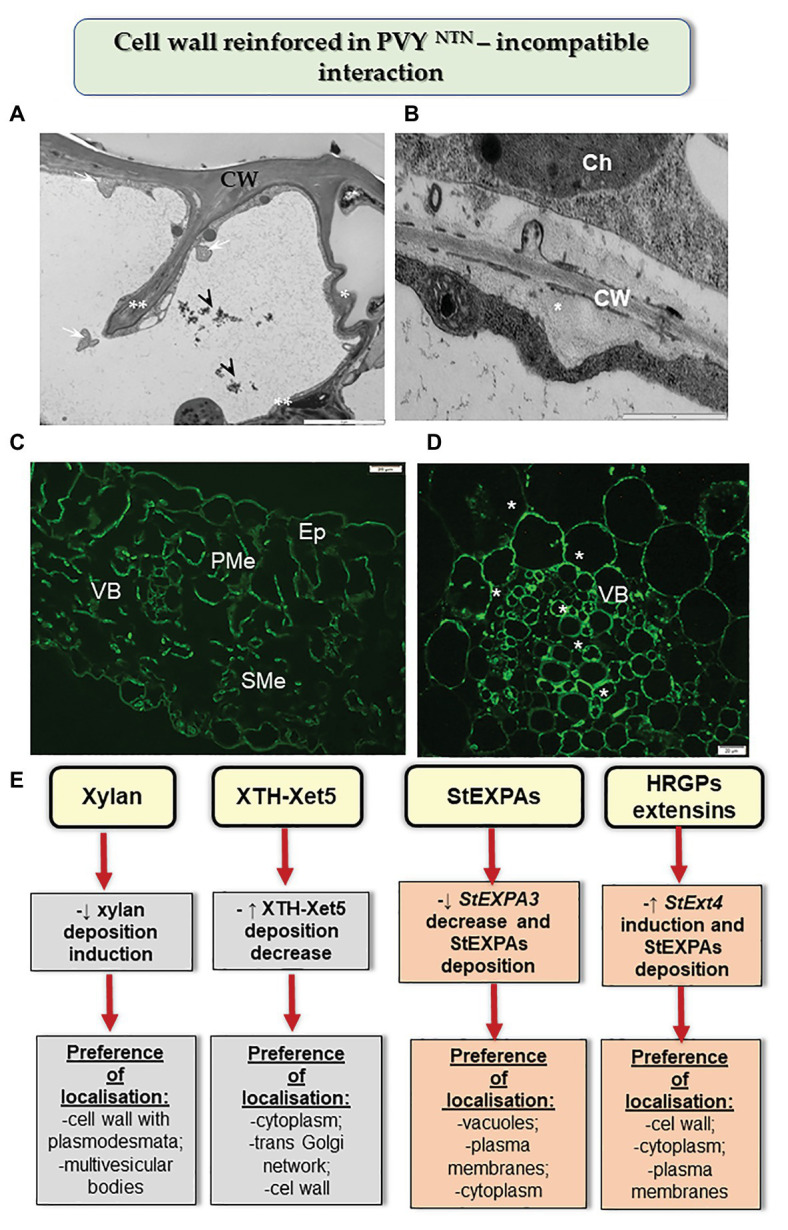
Cell wall reinforced in resistance potato reaction to PVY^NTN^. **(A)** Folded (*) and invaginated cell wall (**) observed in the epidermis during HR. Phenolic compounds (arrowhead) and multivesicular bodies (arrows) found in a vacuole. Bar: 2 μm. **(B)** Deposition of callose material (*) between plasma membrane and cell wall in mesophyll cells during HR. Bar: 1 μm. **(C)** Fluorescence detection of XTH-Xet5 in leaflet tissues after 10 days of PVY inoculation. Bar: 20 μm. **(D)** HRGP extensin signal (*) observed in the vascular bundle at 14 days post inoculation in hypersensitive potato response to PVY^NTN^. Bar: 20 μm. **(E)** Schematic representation of the distribution changes of selected cell wall components in resistance potato response to PVY^NTN^. Ch, chloroplast; CW, cell wall; Ep, epidermis; PMe, palisade mesophyll cells; SMe, spongy mesophyll cells; VB, vascular bundles. (Based on Otulak-Kozieł et al., 2018a,b, 2020, modified).

Moreover, the HR reaction of potato to PVY^NTN^ infection also led to changes in other components of cell walls (Figure 2; Otulak-Kozieł et al., 2018a,b, 2020). This included a decrease in the level of the catalytic subunit of cellulase synthase—CesA4. These changes were more intense than those observed in susceptible potato, and caused rebuilding of cell wall ultrastructure, resulting in a reinforced/thicker cell wall. In many cases of reinforced cell walls, phenolic compounds were also deposited. Otulak-Kozieł et al. (2019) postulated that respiratory burst oxidase homolog D (RbohD) and H_2_O_2_ were vital components of the resistance response in cell wall remodeling in potato-PVY pathosystem. Similarly, the reinforced cell wall and the role of H_2_O_2_ were suggested in the cases of immunity such as the reaction of quinoa to PDV (Kozieł et al., 2020). Along these lines, Zheng et al. (2013) showed that the HR response to RSV was associated with cell wall functions. The authors indicated that changes in peroxidase biosynthesis, glycine-rich cell wall structural protein, cellulose synthase, and XTH/Xet strengthened the physical barriers of rice against RSV. The role of xyloglucans in cell wall remodeling in HR was supported by the results of Otulak-Kozieł et al. (2018b). It was ascertained that during HR, the xylan content decreased but the levels of XTH-Xet5 increased (engaged in xyloglucan metabolism) in cell wall, cytoplasm, and the trans-Golgi network. Therefore, we postulated that the HR activated XTH-Xet5 in areas where xyloglucan endotransglucosylase is synthesized, and later the enzyme was transported to components such as cell wall, cytoplasm, and vacuoles. These findings were similar to those reported by Chen et al. (2016). Hypersensitive reaction is also associated with structural and modified cell wall proteins such as expansins and extensins. Park et al. (2017) showed in *Nicotiana benthamiana* that the absence of expansin A1 (*EXPA1*) gene leads to resistance against TuMV. Otulak-Kozieł et al. (2020) indicated that the *StEXT4* gene is often gradually activated and *StEXPA3* is repressed during HR against PVY^NTN^. They demonstrated that the levels of StEXPAs in cell walls decreased, while the content of HRGPs dynamically increased in the reinforced cell walls; the HRGP extensins accumulated mainly in apoplast, but they were also observed to be deposited in the symplast in the case of resistant plants. Thus, we conclude that changes in the intracellular distribution of HRGPs and StEXPAs are differentially controlled based on the type of PVY^NTN^-potato interactions. These observations confirm that apoplast as well as symplast is involved in defense response mechanisms.

## Future Prospects

Plant cell wall performs a number of important functions in the cell. So far, the structural properties and composition of the cell wall as well as its involvement in the interaction of plants with bacterial and fungal pathogens have been well described. However, modifications occurring in the cell wall during viral infection remain poorly understood. This review presents recent interesting insights into the role the cell wall plays in compatible (susceptible) and incompatible (resistance) reactions. Significant progress has been made in identifying the important components of the cell wall. Results have shown that changes observed in the intracellular dissemination and accumulation of β-1,3 glucanase, components of hemicellulosic cell wall matrix, and cell wall structural and modifying proteins such as expansins, HRGPs, and extensins might be differentially controlled, based on the type of plant-virus interactions, leading to cell wall loosening or the appearance of a reinforced cell wall. This confirms that apoplast as well as symplast is activated as a mechanism of the defense response.

This research topic is promising and provides a better understanding of the multilevel complex network of plant responses to viruses. Further analysis of the functions of specific plant cell components is necessary to establish the regulatory pathways involved in communication between the plant and viral pathogen. This can be possible with the use of tools such as atomic force microscopy (AFM). AFM images can show, at the nanometer scale, the heterogeneity of the cell wall as well as the spatial distribution of both soft and rigid polymers of the cell wall matrix (Gierlinger, 2018). AFM can be employed together with electron tomography to study the cell wall in mutants, for example, of cellulose synthase in the infected plants. Extensive computational simulations of mechanical properties may reveal the three-dimensional organization of the cell wall during interactions (Otegui and Pennington, 2019). Furthermore, spectroscopic techniques (such as confocal Raman spectroscopy) may non-disruptively indicate the cell wall structures and also be applied as a high-throughput method to describe the phenotypes of cell wall mutants. Transgenic/mutant plants are formed as a result of the generation of resistance to plant pathogens based on changes in cell wall-associated elements (Moscetti et al., 2013; Ferrari et al., 2014; Lionetti et al., 2014; Woo et al., 2014). Insertion of polygalacturonase-inhibiting proteins from bean and overexpression of natural wheat xylanase inhibitor TAXI-III induced resistance in wheat against *Fusarium graminearum* (Moscetti et al., 2013; Ferrari et al., 2014). Mutants or overexpression lines with lignin modification have been analyzed by many researchers, and they seem to play an important role in the pathogen resistance—as a passive or active regulatory component of immune response, such as in the case of cotton resistance to wilt (*Verticillium dahliae*) as presented by Xu et al. (2011) and Shi et al. (2012). Several examples of cell wall change-mediated immune reaction in genetically engineered plants have been reviewed in detail by Miedes et al. (2014), Smirnova and Kochotev (2016), and Bacete et al. (2018). The promising techniques for the generation of cell wall mutants include the T-DNA insertions, TILLING lines, and especially CRISPR/Cas9 lines, where crucial cell wall components and genes are blocked or overexpressed. In particular, the CRISPR/Cas9 system may provide an insight into the genetic control of the structure and functions of plant cell wall (Whitehead et al., 2018; Cao et al., 2020; McCarty et al., 2020). At present, these methods are generally used in studies focusing on cell wall synthesis pathways (including enzymes) and analyzing the exact functions of cell wall elements (Zhang and Showalter, 2020) for the improvement of crop products (Wang C. et al., 2019; Wang D. et al., 2019). However, Makarova et al. (2018) indicated the application of the CRISPR/Cas9 system for generating plant resistance (not precisely associated with cell wall) against various pathogens. Such studies can allow for a better understanding of the processes involved in the communication of the plant cell with viruses.

## Author Contributions

EK and KO-K conceived the project, analyzed data, and participated in writing the manuscript. JB analyzed data and helped with critical comments of the manuscript. All authors contributed to the article and approved the submitted version.

### Conflict of Interest

The authors declare that the research was conducted in the absence of any commercial or financial relationships that could be construed as a potential conflict of interest.
